# Unexplained Cardiac Uptake on ^99m^Tc-MDP Bone Scan in a Patient with Prostate Cancer

**DOI:** 10.22038/aojnmb.2025.85251.1609

**Published:** 2025

**Authors:** Malik E. Juweid, Baraa Alsyouf, Nour Kasasbeh, Waleed Mahafza, Serin Moghrabi, Hanna Al-Makhamreh, Akram Saleh

**Affiliations:** 1 Division of Nuclear Medicine, Department of Radiology and Nuclear Medicine, University of Jordan, Amman, Jordan; 2 Department of Radiology and Nuclear Medicine, University of Jordan, Amman, Jordan; 3 Department of Nuclear Medicine, King Hussein Cancer Center, Amman, Jordan; 4 Division of Cardiology, Department of Internal Medicine, University of Jordan, Amman, Jordan

**Keywords:** Cardiac Uptake, ^99m^Tc-MDP, Bone Scan, Prostate Cancer

## Abstract

This case report presents a case of unusual diffuse cardiac uptake (Peruguni 3: uptake greater than rib uptake) on a ^99m^Tc-methylene diphosphonate bone scan in an 83-year-old patient with metastatic prostate cancer which is almost resolved (Peruguni 1: uptake less than rib uptake) on a follow up bone scan about 4.4 months later. Laboratory values and imaging were negative for cardiac amyloidosis and a thorough review of the patient's medical chart did not reveal any other possible causes, pharmacologic or otherwise, thus deeming the uptake non-specific. While increased non-specific cardiac ^99m^Tc-diphosphanate uptake has been previously reported in elderly prostate cancer patients, possibly attributable to asymptomatic atherosclerosis, this explanation is unlikely considering that the uptake almost resolved within a relatively short period of time. It is clinically important to rule out amyloidosis in patients with increased cardiac uptake on bone scans. However, we believe that clinicians should also consider the possibility of non-specific uptake as a cause for cardiac uptake on bone scan, which would only require follow-up rather than medical intervention.

## Introduction

Multiple technetium-99m (^99m^Tc) labeled phosphate analogs are used as radio-pharmaceuticals in bone scintigraphy, including methylene diphosphonate (MDP), 3,3-diphosphono-1,2-propanodicarboxylic acid (DPD), pyrophosphate (PYP) and hydroxyl-methylene diphosphonate (HDMP) (1). These compounds function by binding to hydroxy-apatite crystals in bone through a process known as chemisorption, which reflects osteoblastic activity and skeletal metabolism (2).

 Moreover, some of these radiotracers have uses beyond imaging bone. In particular, ^99m^Tc-PYP, ^99m^Tc-DPD and ^99m^Tc-HMDP have been used for the diagnosis and prognosis of cardiac transthyretin amyloidosis (ATTR) while ATTR is only rarely positive using ^99m^Tc-methylene diphosphonate (MDP) (3–6). The mechanism of uptake of phosphate radiolabeled analogs in ATTR amyloidosis is not yet completely understood, but some authors attribute it to the rich calcium content seen in ATTR (7, 8). However, this does not explain why ATTR is usually negative using ^99m^Tc-MDP (6). 

 Importantly, such uptake is not specific for ATTR, regardless of the tracer used as it may also be observed in conditions like immuno-globulin light chain (AL) amyloidosis, prior myocardial infarction, or overlying rib fractures (3).

 Increased cardiac uptake of ^99m^Tc-MDP has been reported to be more frequent in elderly prostate cancer patients, possibly due to the widespread use of this tracer for bone scintigraphy in these patients to detect osteoblastic metastases and facilitate staging, restaging, therapy assessment, and treatment planning (9-11). 

 We present a case of a patient who exhibited diffuse cardiac uptake on a ^99m^Tc-MDP bone scan without an identifiable cause.

## Case Presentation

 This case presents an 83-year-old male with a history of hypertension and ischemic heart disease post coronary artery bypass graft in 1997. His regular medications included amlodipine 5 mg once per day (QD), valsartan 160 mg QD, indapamide 1.5 mg QD, metoprolol succinate 50 mg QD, rosuvastatin 10 mg QD, and clopidogrel 75 mg QD. He had never undergone any nuclear medicine scan, and he was never offered any myocardial perfusion scan by his treating physicians. In 2024, the patient was diagnosed with metastatic acinar adeno-carcinoma of the prostate (cribriform pattern, grade 4 with a Gleason score of 4+4) with a baseline prostate-specific agent (PSA) level of 3.1 ng/mL. His initial TNM staging was cT1cNxM1c according to the American Joint Committee on Cancer (12). He started Goserelin acetate 10.8 mg by intramuscular injection once (every 3 months) two weeks prior to his initial bone scan.

**Figure 1:**
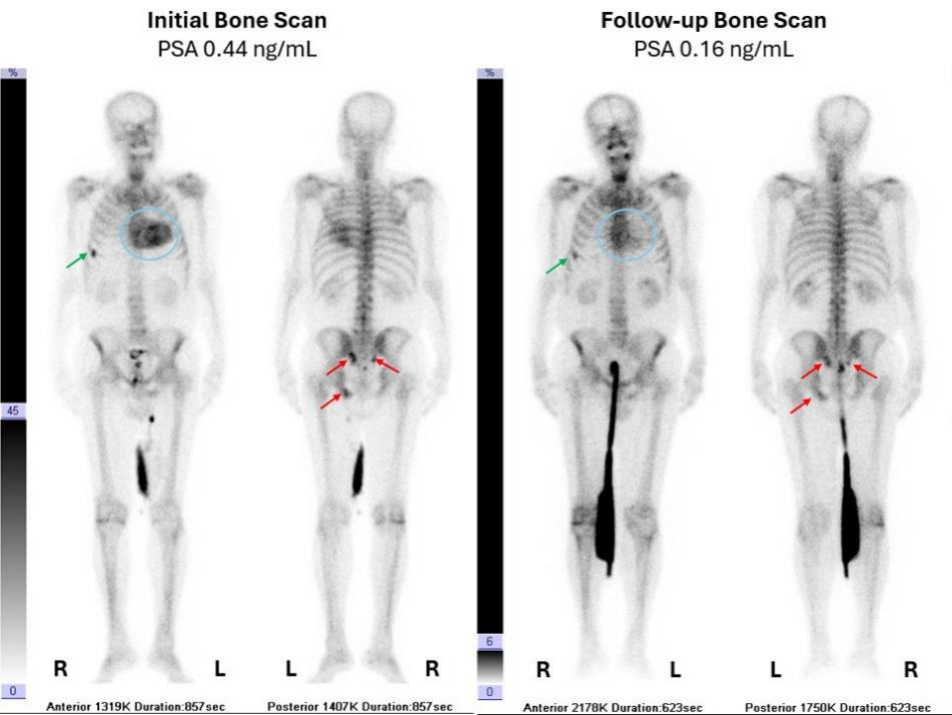
. Left: baseline ^99m^Tc-MDP bone scan which shows intense cardiac uptake (Peruguni 3: uptake greater than rib uptake) (**blue circle**) and a likely post-traumatic focus in the right 6^th^ rib (**green arrow**), as well as a few suspicious metastatic lesions (**red arrows**). Right**:** follow-up bone scan with ^99m^Tc-MDP, performed 132 days after the initial bone scan, showing only minimal cardiac uptake (Peruguni 1: uptake less than rib uptake) (**blue circle**) as well as decreased uptake in the traumatic (**green arrow**) and suspicious metastatic lesions (**red arrows**)

**Figure 2:**
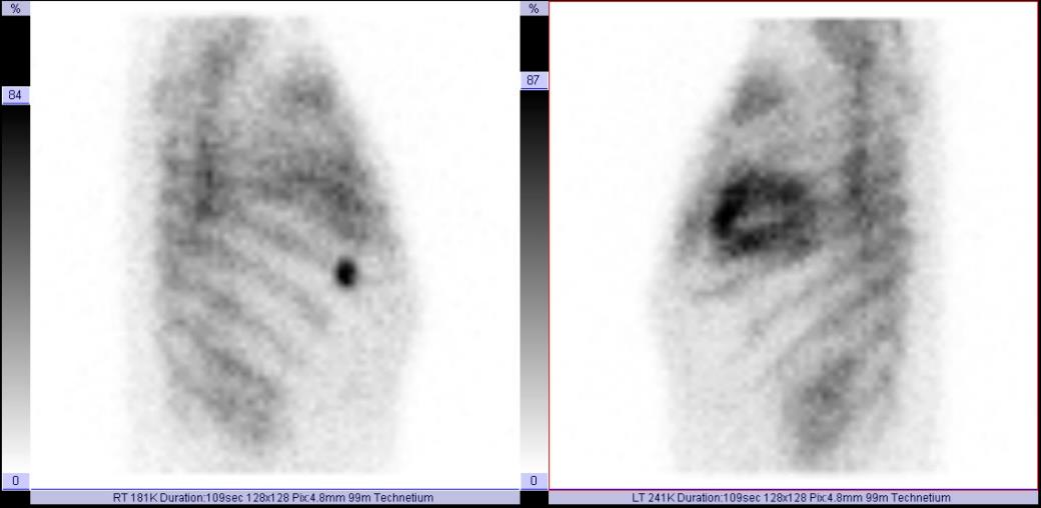
Lateral chest images of the baseline ^99m^Tc-MDP bone scan showing intense cardiac uptake (Peruguni 3: uptake greater than rib uptake)

 For his initial bone scintigraphy, he was imaged after the administration of 15 mCi of ^99m^Tc-MDP approximately 3 hours after the time of injection. The scan showed suspicious lesions in the left ischium and bilateral sacroiliac regions, a likely post-traumatic focus in the right 6th rib and intense diffuse cardiac uptake (Peruguni 3: uptake greater than rib uptake) as shown in Figure 1, left and Figure 2. No 24-hour delayed bone scan was performed. Cardiac amyloidosis was suspected. The tumor board in consultation with the treating cardiologist decided to investigate the possibility of cardiac amyloidosis further. The board also decided to add Enzalutamide (160 mg QD) two weeks later due to the presence of bone metastases.

 The patient reported no symptoms of active ischemic heart disease or heart failure. Echocardiography, cardiac magnetic resonance, serum, and urine immunofixation tests were negative for cardiac amyloidosis. A follow-up scan with the same dose and time interval after injection was performed after 132 days (~4.4 months), and it showed improved bony metastases without any new lesions and now only minimal cardiac uptake (Peruguni 1: uptake less than rib uptake) (Figure 1, right). Apart from a drop in PSA level from 0.44 ng/mL at the time of the initial bone scan to 0.16 ng/mL at the time of the follow-up bone scan, all the patients’ biochemical markers were stable and within normal limits at the time of both scans. 

 Considering the lack of an obvious cause for the initial cardiac uptake and its significant decrease over a few months, it was assumed that it was non-specific uptake and likely benign in etiology.

## Discussion

 This report presents a particularly interesting case, not only because of marked cardiac uptake on the initial ^99m^Tc-MDP bone scan decreasing significantly in 4.4 months, but also because no obvious cause for the initial or decreasing uptake could be identified.

 Most cardiac amyloidosis cases belong to either the AL or ATTR subtypes, the latter of which is strongly associated with cardiac uptake on bone scintigraphy using ^99m^Tc-PYP, ^99m^Tc-DPD, and ^99m^Tc-HMDP but not ^99m^Tc-MDP (2, 3). In addition to the causes of cardiac uptake without evidence of ATTR or AL amyloidosis mentioned in the introduction, cardiac uptake may also be due to myocardial ischemia, inflammatory myocarditis or pericarditis, hypercalcemia, secondary hyperparathyroidism, prior hydroxychloroquine and Adriamycin administration and radiation therapy (1,3, 13, 14). None of these potential causes were present in our case. Chadrawar et al (15) also speculated about the possible role of statins in myocardial uptake due to their potential side effects of statin-induced myositis or rhabdo-myolysis (16, 17). However, to the best of our knowledge, all reported cases were found in skeletal muscles, and none have reported uptake in the myocardium, making it unlikely that this may be the cause in our patient who did not complain of muscle pain nor had any abnormal biochemical findings to indicate such a condition.

 Interestingly, increased ^99m^Tc-diphosphanate uptake in the heart has been reported more frequently in elderly prostate cancer patients without an obvious cause (9, 10) although in one report it was attributable to asymptomatic atherosclerosis and deemed of little relevance in the absence of cardiac symptoms (11). 

 Moreover, one report noted that this benign uptake was more commonly observed in patients above the age of 80 years and was more commonly observed with HDP than MDP (14). 

 In our case, atherosclerosis is an unlikely cause as the uptake resolved within a relatively short period of time. Finally, it is extremely unlikely that the diffuse cardiac uptake seen initially was due to diffuse myocardial metastases from prostate cancer that were eliminated by hormonal therapy, as cardiac involvement by prostate cancer is exceedingly rare, comprising less than 0.016% of all myocardial metastases and when occurring it is rather focal than diffuse (15, 18, 19).

 In our case, we have not employed 24-hour delayed imaging, which could have resulted in resolution of the myocardial uptake on the initial scan, as has been reported by Demirel et al (20) in a patient with multiple myeloma where diffuse myocardial uptake noted on the 3-hour ^99m^Tc-HDP scan disappeared on the 24-hour scan. However, the disappearance of uptake at 24 hours does not necessarily mean that this uptake is non-specific and excluding cardiac amyloidosis. In fact, Demirel et al (20) still presumed that the cardiac uptake was related to AL associated with multiple myeloma and did not have an explanation of why the uptake disappeared on delayed imaging. To our knowledge, there are no other reports of 24-hour imaging related to cardiac uptake on bone scans.

 In conclusion, despite the non-specific ^99m^Tc-MDP cardiac uptake in our case, investigating such uptake is important as it may represent cardiac amyloidosis necessitating different management. If these investigations fail to identify a cause of cardiac uptake, it should be deemed nonspecific and would only require follow-up.
